# The Perception of Rhizosphere Bacterial Communication Signals Leads to Transcriptome Reprogramming in *Lysobacter capsici* AZ78, a Plant Beneficial Bacterium

**DOI:** 10.3389/fmicb.2021.725403

**Published:** 2021-08-18

**Authors:** Ana Bejarano, Michele Perazzolli, Ilaria Pertot, Gerardo Puopolo

**Affiliations:** ^1^Center of Agriculture, Food, Environment, University of Trento, San Michele all’Adige, Italy; ^2^Department of Sustainable Agro-Ecosystems and Bioresources, Research and Innovation Centre, Fondazione Edmund Mach, San Michele all’Adige, Italy

**Keywords:** diffusible communication signals, rhizosphere, *Lysobacter*, bacterial interactions, transcriptome

## Abstract

The rhizosphere is a dynamic region governed by complex microbial interactions where diffusible communication signals produced by bacteria continuously shape the gene expression patterns of individual species and regulate fundamental traits for adaptation to the rhizosphere environment. *Lysobacter* spp. are common bacterial inhabitants of the rhizosphere and have been frequently associated with soil disease suppressiveness. However, little is known about their ecology and how diffusible communication signals might affect their behavior in the rhizosphere. To shed light on the aspects determining rhizosphere competence and functioning of *Lysobacter* spp., we carried out a functional and transcriptome analysis on the plant beneficial bacterium *Lysobacter capsici* AZ78 (AZ78) grown in the presence of the most common diffusible communication signals released by rhizosphere bacteria. Mining the genome of AZ78 and other *Lysobacter* spp. showed that *Lysobacter* spp. share genes involved in the production and perception of diffusible signal factors, indole, diffusible factors, and *N*-acyl-homoserine lactones. Most of the tested diffusible communication signals (i.e., indole and glyoxylic acid) influenced the ability of AZ78 to inhibit the growth of the phytopathogenic oomycete *Pythium ultimum* and the Gram-positive bacterium *Rhodococcus fascians*. Moreover, RNA-Seq analysis revealed that nearly 21% of all genes in AZ78 genome were modulated by diffusible communication signals. 13-Methyltetradecanoic acid, glyoxylic acid, and 2,3-butanedione positively influenced the expression of genes related to type IV pilus, which might enable AZ78 to rapidly colonize the rhizosphere. Moreover, glyoxylic acid and 2,3-butanedione downregulated tRNA genes, possibly as a result of the elicitation of biological stress responses. On its behalf, indole downregulated genes related to type IV pilus and the heat-stable antifungal factor, which might result in impairment of twitching motility and antibiotic production in AZ78. These results show that diffusible communication signals may affect the ecology of *Lysobacter* spp. in the rhizosphere and suggest that diffusible communication signals might be used to foster rhizosphere colonization and functioning of plant beneficial bacteria belonging to the genus *Lysobacter*.

## Introduction

The soil is one of the largest microbial reservoirs on Earth, where millions of bacteria and fungi, and less frequently archaea, algae, and protozoa, interact with each other and the plants. In particular, the rhizosphere, the soil compartment influenced by the root exudates, is a hot spot of microbes, as root exudates are a major carbon source for soil microorganisms and a driving force of their population density and activities (Raaijmakers et al., 2009).

*Lysobacter* spp. belonging to the Xanthomonadaceae family are commonly found in agricultural soils (Lee et al., 2006; Postma et al., 2008; Choi et al., 2014) and especially in the plant rhizosphere. Indeed, they have been found in high abundance in the rhizosphere of maize (Schmalenberger and Tebbe, 2003; García-Salamanca et al., 2013), potato (Van Overbeek and Van Elsas, 2008; Turnbull et al., 2012), soybean (Liang et al., 2014), switchgrass (Rodrigues et al., 2018), and common bean (Pérez-Jaramillo et al., 2019). In terms of ecosystem, several studies have associated *Lysobacter* spp. with the phenomenon of soil disease suppressiveness (Puopolo et al., 2018). Several studies even showed that the application of *Lysobacter* spp. reduced diseases caused by different phythopatogenic microorganisms in several crops (Ji et al., 2008; Ko et al., 2009; Puopolo et al., 2010). Yet the sole application of *Lysobacter* spp. in soils did not always lead to an effective control of phytopathogenic microorganisms probably due to the need to interact with a specific microbial community to become rhizosphere competent (Postma et al., 2009; Gómez Expósito et al., 2015).

Microbial intra- and interspecies signaling occurring *via* diffusible communication signals plays an important role in soil microbial interactions (Abisado et al., 2018). Diffusible communication signals coordinate interactions both within a species and between species and modulate microbial physiological traits such as motility, attachment, biofilm formation, and biosynthesis of secondary metabolites (Venturi and Keel, 2016). *N*-acyl-homoserine lactones (AHLs), produced by Gram-negative plant-associated bacteria, are among the most studied diffusible communication signals (Case et al., 2008; Papenfort and Bassler, 2016). Several works showed that endogenous and exogenous AHLs play an essential role in multiple bacterial physiological and biochemical behaviors (Stephens and Bentley, 2020) and can even act as interkingdom signals (Venturi and Fuqua, 2013). Diffusible signal factors (DSFs) are another subgroup of diffusible communication signals produced by Gram-negative bacteria; and they have been linked to virulence, motility, biofilm production, and extracellular enzyme production (Ryan et al., 2015). Xanthomonadaceae family also use diffusible factors (DFs) as diffusible communication signals; these signals are involved in the regulation of secondary metabolites biosynthesis and antioxidant activity (He et al., 2011; Qian et al., 2013; Zhou et al., 2013). In addition, plant-associated bacteria produce volatile organic compounds (VOCs) that are involved in communication and competition between physically separated soil microorganisms (Schmidt et al., 2015). Among VOCs, indole (IND) is a ubiquitous interkingdom signal that influences antibiotic resistance, motility, biofilm formation, and virulence and has the potential to be a diffusible communication signal (Lee and Lee, 2010). 2,3-Butanedione (BUT) and glyoxylic acid (GLY) are other VOCs mediating changes in gene expression related to motility and antibiotic resistance (Kim et al., 2013).

Based on this body of knowledge, it is conceivable that when *Lysobacter* spp. are applied to the rhizosphere, they will firstly perceive diffusible communication signals produced by the indigenous microbial community before establishing any physical interaction with other rhizosphere-associated microorganisms. The perception of these diffusible communication signals might lead to changes in their transcriptome, which in turn might ultimately lead to changes in *Lysobacter* spp. rhizosphere competence and their ability to control plant pathogens. Indeed, it has been shown that DSFs, DFs, and IND regulate the biosynthesis of the heat-stable antifungal factor (HSAF), a potent antifungal compound, and twitching motility in *Lysobacter enzymogenes* (Qian et al., 2013; Han et al., 2017; Su et al., 2017; Feng et al., 2019). However, with the only exception of the involvement of DSFs and AHLs in *Lysobacter brunescens* behavior (Ling et al., 2019a,b), a complete overview of the overall effect of diffusible communication signals in the ecology of *Lysobacter* spp. in the rhizosphere has not been described yet.

In this regard, we aimed at unveiling the response of *Lysobacter* spp. to diffusible communication signals. To that end, we used *Lysobacter capsici* AZ78 (AZ78), a model plant beneficial bacterium isolated from the rhizosphere of tobacco plants (Puopolo et al., 2014) provided with physiological fundamental traits to survive in the rhizosphere (Brescia et al., 2020). Firstly, gene-encoding proteins involved in cell–cell communication systems were identified by genome mining. Next, we carried out functional experiments aimed at assessing changes in AZ78 cell growth and antimicrobial activity upon exposure to 13-methyltetradecanoic acid (*L. enzymogenes* DSF-like molecule, LeDSF3, Han et al., 2015), IND, GLY, BUT, 3-hydroxybenzoic acid (3HBA), 4-hydroxybenzoic acid (4BHA), *N*-(3-hexanoyl)-L-homoserine lactone, *N*-(3-oxooctanoyl)-L-homoserine lactone, and *N*-(3-oxododecanoyl)-L-homoserine lactone. Simultaneously, gene expression profiling of AZ78 exposed to the above-mentioned diffusible communication signals was carried out by high-throughput RNA-Seq.

## Materials and Methods

### Microorganisms and Diffusible Communication Signals

Bacterial strains (Supplementary Table 1) were routinely grown on Nutrient Agar (NA; Oxoid, Basingstoke, United Kingdom) at 27°C. The phytopathogenic oomycete *Pythium ultimum* was maintained on Potato Dextrose Agar (Oxoid) at 25°C.

LeDSF3 was obtained from Avanti Polar Lipids (Alabaster, AL, United States). IND, GLY, BUT, 3HBA, 4BHA, *N*-(3-hexanoyl)-L-homoserine lactone, *N*-(3-oxooctanoyl)-L-homoserine lactone, and *N*-(3-oxododecanoyl)-L-homoserine lactone were purchased from Merck (Sigma-Aldrich, Darmstadt, Germany). Aqueous stock solutions were prepared, except for LeDSF3 and the mixture of AHLs, which were prepared in pure methanol.

### Genome Mining

AZ78 genome was mined to identify putative genes involved in cell–cell communication systems using nucleotide and protein sequence comparison. Genes from *L. enzymogenes* C3, *Stenotrophomonas maltophilia* (*Sm*) K279a, and *Xanthomonas campestris* pv. *campestris* (*Xcc*) ATCC 33913^*T*^ were aligned against AZ78 genome, using RAST (Aziz et al., 2008) to identify putative AZ78 genes responsible for diffusible communication signal synthesis, reception, and regulation using a cut-off of 1 × 10^–5^ at amino acid level. Putative genes were analyzed with BLASTP (Johnson et al., 2008), and length >70 and identity >70% at amino acid level were used as threshold. Identified gene clusters encoding putative proteins involved in cell–cell communication systems in AZ78 were then used to mine the *Lysobacter* spp. genomes, following the methodology described above. All genomes were downloaded from the National Center for Biotechnology Information (NCBI)^1^ (Supplementary Table 1). For the phylogenetic analyses, nucleotide sequences were aligned using ClustalW (Thompson et al., 1994). Evolutionary distances were assessed by applying Kimura’s two-parameter model (Kimura, 2020); and the best phylogenetic trees were inferred by neighbor-joining method (Saitou and Nei, 1987) implemented in MEGA 7 (Kumar et al., 2016). Confidence values for nodes in the trees were generated by bootstrap analysis (Felsenstein, 1985) using 1,000 permutations of the data sets.

### Assessment of Diffusible Communication Signal Production

Production of AHLs by AZ78 and *Lysobacter* spp. type strains was assessed by evaluating their ability to restore violacein production in *Chromobacterium violaceum* CV026 and/or to promote *lacZ* transcription in *Agrobacterium tumefaciens* NT1 (pZLR4) as previously described (Cha et al., 1998; Steindler and Venturi, 2007). In brief, candidate strains were grown on NA close to each reporter strain to form a “T,” and the phenotypic change associated with the presence of AHLs was observed as a gradient with the most response observed at the meeting point of the two strains. Medium used in assays involving *A. tumefaciens* was supplemented with 1.6 μg/ml of X-Gal (5-bromo-4-chloro-3-indolyl β-D-galactopyranoside, Sigma-Aldrich). Likewise, the ability to release DSF was determined using the bacterial reporter strain *Xcc* 8523 pL6engGUS according to Slater et al. (2000). Briefly, *Xcc* 8523 pL6engGUS was grown in 10 ml of NYG (5 g/L of peptone, 3 g/L of yeast extract, and 20 g/L of glycerol) supplemented with 10 μg/ml of tetracycline to an optical density (OD_600_) of 0.7. Cells were harvested by centrifugation and reconstituted in 1 ml of fresh NYG, added to 100 ml of cold NGY containing 1% BD Difco Noble Agar (BD Biosciences, Sparks, MD, United States), supplemented with 80 μg/ml of X-Glu (5-bromo-4-chloro-3-indolyl β-D-glucuronide sodium salt; Sigma-Aldrich), and plated into petri plates. Candidate strains were then pin inoculated and incubated for 48 h at 27°C. The presence of a blue halo around the colony indicated DSF activity. *Pseudomonas chlororaphis* M71 (Puopolo et al., 2011) was used as an AHL-positive control, whereas *Xcc* 8004 was used as a DSF positive control (Barber et al., 1997). For each condition, five replicates were used, and the experiment was repeated.

### Effect of Diffusible Communication Signals on Antimicrobial Activity

The effect of diffusible communication signals on AZ78 antimicrobial activity was evaluated on Rhizosphere Mimicking Agar (RMA) (Brescia et al., 2020). At first, preliminary experiments where AZ78 was grown on RMA amended with different concentrations of the selected compounds were carried out to select minimum effective concentrations—the lowest concentrations showing the highest effect—of diffusible communication signals. Thereafter, the final experimental design was made up of eight treatments (Supplementary Table 2). Inhibitory activity of AZ78 against *P. ultimum* was evaluated by using the classic dual-culture method as previously described (Puopolo et al., 2016). In brief, 10 μl of AZ78 cell suspension (1 × 10^8^ CFU/ml) were spot-inoculated at 3 cm of the edge of a plate. After 48-h incubation at 27°C, mycelium plugs (4 mm) were cut from the edge of 1-week-old *P. ultimum* plate, placed at 2.5-cm distance from AZ78, and incubated at 25°C for 168 h. AZ78 activity against *Rhodococcus fascians* LMG 3605 was determined by spot-inoculating 10 μl of AZ78 cell suspension (1 × 10^8^ CFU/ml) in the center of an RMA plate (Puopolo et al., 2016). After 48-h incubation at 27°C, AZ78 cells were killed by exposure to chloroform vapor for 60 min (Puopolo et al., 2016). Dishes were aerated under a laminar flow for 60 min, overlaid with 4 ml of 0.45% agar phosphate-buffered saline (PBS) containing *R. fascians* LMG 3605 (1 × 10^7^ CFU/ml) and incubated at 27°C for 72 h. RMA dishes seeded only with *P. ultimum* or *R. fascians* LMG 3605 were used as control.

Pictures were obtained with Bio-Rad Quantity One software implemented in a Bio-Rad GelDoc Imaging system (Bio-Rad Laboratories, Hercules, CA, United States). Inhibitory activity was quantified by scoring *P. ultimum* or *R. fascians* LMG 3605 growth area (cm^2^) using ImageJ 1.52a (Schneider et al., 2012) and calculated according to the formulas below:

Antioomycete activity(%)=(Inhibition of mycelial growth in presence of Diffusible Communication Signals Inhibition of mycelial growth in absence of Diffusible Communication Signals -1)×100

where

Inhibition of mycelial growth =(1-Mycelium area in presence of L capsici AZ78 Mycelium area in absence of L capsici AZ78 )×100

The effect of diffusible communication signals on AZ78 antibacterial activity was assessed as follows:

Antibacterial activity (%)=(Inhibition zone area in presence of Diffusible Communication Signals Inhibition zone area in absence of Diffusible Communication Signals -1)×100

In all cases, treatments included five replicates, and experiments were repeated.

### Evaluation of Diffusible Communication Signal Effect on Cell Growth

The effect of diffusible communication signals on AZ78 cell growth rate was assessed on 1/10 Tryptic Soy Broth (Oxoid) amended with each diffusible communication signal (Supplementary Table 2). AZ78 (starting concentration 1 × 10^7^ CFU/ml) was grown at 27°C on a 96-well plate (200 μl), and absorbance at 600 mm was recorded on a microplate reader (Synergy 2 Multi-Mode Microplate Reader, BioTek, Winooski, VT, United States). Non-inoculated media were used as blank. For each condition, five replicates were used. The experiment was repeated.

### RNA Extraction

The AZ78 response to diffusible communication signals was evaluated on RMA, and the experimental design was made up of eight treatments in triplicate (Supplementary Table 2). Ten microliters of AZ78 cell suspension (1 × 10^10^ CFU/ml) were spot-inoculated in the center of an RMA plate and incubated at 27°C for 48 h. Plugs (7-mm diameter) were collected from the AZ78 macrocolonies, immediately frozen in liquid nitrogen, and stored at −80°C. Frozen samples were processed according to Brescia et al. (2020), and total RNA was extracted using Spectrum Plant Total RNA Kit (Sigma-Aldrich). DNase treatment was performed with the RNase-Free DNase set (Qiagen, Hilden, Germany). RNA integrity and concentration were assessed using a 2200 TapeStation System (Agilent Technologies, Santa Clara, CA, United States) and a Qubit 4 Fluorometer (Thermo Fisher Scientific, Carlsbad, CA, United States) with Qubit RNA BR assay kit (Thermo Fisher Scientific), respectively (Supplementary Table 3).

### Illumina Sequencing and Mapping to the Reference Genomes

Library construction and Illumina Sequencing were carried out at Fasteris (Plan-les-Ouates, Switzerland). Ribosomal RNA (rRNA) depletion was performed using the Ribo-Zero rRNA Removal Kits (Bacteria) (Illumina, San Diego, CA, United States). Complementary DNA (cDNA) libraries were synthesized using TruSeq Stranded mRNA Library Prep (Illumina, United States); they were multiplexed (two libraries per lane); and paired-end reads of 150 nucleotides were obtained using an Illumina HiSeq 4000 (Illumina), resulting in ∼7–42 million reads per sample (Supplementary Table 4). Raw sequences were deposited at the Sequence Read Archive of the NCBI under BioProject number PRJNA714393.

Sequence analysis was carried out using Omicsbox 1.3.11.^2^ Illumina HiSeq data were assessed for quality using FastQC (Andrews, 2010). Raw reads for each sample were trimmed to increase overall quality using Trimmomatic 0.38 (Bolger et al., 2014). The resulting reads were aligned to AZ78 genome (Supplementary Table 1) using the STAR 2.7.5a (Dobin et al., 2013), and read counts were extracted from STAR alignments using HTSeq (Anders et al., 2015).

### Identification of Differentially Expressed Genes and Functional Annotation of RNA-Seq

Genes with zero counts in all replicates were excluded from the analysis, and raw counts were normalized using the trimmed mean of *M*-values method (Robinson and Oshlack, 2010). Differentially expressed genes (DEGs) were identified using edgeR 3.28.0 (Robinson et al., 2010) using a *p*-value < 0.01 and a log fold change (FC) of at least onefold upregulation/downregulation as cut-off values. Venn diagrams summarizing DEG distribution were drawn with VennPainter (Lin et al., 2016). Hierarchical clustering and heatmaps were created with TreView3 (Saldanha, 2004).

The protein sequences of all predicted genes (Puopolo et al., 2016) were functionally annotated using Blast2Go^3^ (Conesa et al., 2005). Default settings were applied, and a minimum *E*-value of 10^–5^ was imposed as cut-off. DEGs were further annotated based on the NCBI gene description and classified in 20 functional categories.

### Validation of RNA-Seq

First-strand cDNA was synthetized from 600 ng of purified RNA with SuperScript III Reverse Transcriptase (Invitrogen, Carlsbad, CA, United States) using random hexamers, according to manufacturer’s instructions. qRT-PCRs were carried out with Platinium SYBR Green qPCR Super-Mix-UDG (Invitrogen, United States), and specific primers (Supplementary Table 5) were designed using Primer3 software (Untergasser et al., 2012). Primer specificity was assessed using PCR before gene expression analysis. qRT-PCRs were run for 50 cycles (95°C for 15 s and 60°C for 45 s) on a LightCycler 480 (Roche Diagnostics, Mannheim, Germany). Each sample was examined in three technical replicates, and dissociation curves were analyzed to verify the specificity of each amplification reaction. LightCycler 480 software, version 1.5 (Roche Diagnostics, Mannheim, Germany) was used to extract cycle threshold (Ct) values based on the second derivative calculation; and the LinReg software, version 11.0, was used to calculate reaction efficiencies for each primer pair (Ruijter et al., 2009). Relative expression levels were calculated according to the Pfaffl equation (Pfaffl, 2001) using AZ78 growing in RMA as calibrator. The housekeeping gene *recA* (AZ78_1089; Puopolo et al., 2016) was used as constitutive gene for normalization, as its expression was not significantly affected by growth media and conditions (Tomada et al., 2016, 2017; Brescia et al., 2020). The linear relationship between the RNA-Seq log_2_FC values and the qRT-PCR log_2_FC values of selected genes was estimated by Pearson’s correlation analysis.

### Statistical Analysis

Percentage values were arcsine square root transformed to normalize distributions and to equalize variances. Comparisons between repeated experiments of antimicrobial activity were done using two-way analysis of variance (ANOVA), and the data were pooled when no significant differences were found according to the *F*-test (*p* > 0.05). Data were analyzed using one-way ANOVA, and Tukey’s test (α = 0.05) was used to detect significant differences. Statistical analyses were carried out using IBM SPSS Statistics for Windows, version 21.0 (IBM Corp, Armonk, NY, United States).

## Results

### Cell–Cell Communication Systems in *Lysobacter capsici* AZ78 Genome

Putative *rpf* genes were found in the AZ78 genome (Table 1 and Supplementary Figures 1, 2). The *rpfF/rpfC* region (3,947,548–3,948,450 bp) was located far from the *rpfG/rpfB* region (857,114–859,152 bp) in AZ78 (Supplementary Figure 3a). DSF biosynthesis was confirmed by AZ78 ability to induce the glucuronidase activity in *Xcc* 8523 pL6engGUS like the control strain *Xcc* 8004 (Supplementary Figure 3b). As for VOCs, putative gene-encoding IND synthase and QseB/QseC system were found in AZ78 genome and in other *Lysobacter* spp. (Table 1 and Supplementary Figure 4). Homologues of the chorismatase needed for DF production and the LysR family transcription factor involved in the DF regulatory cascade were also found in AZ78 genome (Table 1 and Supplementary Figure 5). *luxR* gene, responsible for the detection and response to AHLs, was also present in AZ78 (Table 1 and Supplementary Figure 5). LuxR was not associated with its cognate AHL synthase (LuxI) in AZ78 or in other *Lysobacter* species, with only exception of *Lysobacter daejeonensis* GH1-9^*T*^ having a *luxI* homologue. The absence of LuxI homologs was confirmed by the inability of AZ78 to restore β-galactosidase activity and violacein production in the reporter strains *A. tumefaciens* NT1 pZRL4 and *C. violaceum* CV026, respectively (Supplementary Figure 6). In contrast, *L. daejeonensis* GH1-9^*T*^ was able to restore violacein production in *C. violaceum* CV026, confirming the relation between the presence of *luxI* and AHL production.

**TABLE 1:** Cell–cell communication genes present in the genome of *Lysobacter capsici* AZ78.

Gene abbreviation	*L. capsici* AZ78	Annotation in *L. capsici* AZ78	*Lysobacter enzymogenes* C3		*Stenotrophomonas maltophilia* K279a		*Xanthomonas campestris* pv. *campestris* ATCC 33913^*T*^	
*rpfG*	AZ78_0630	Response regulator	GLE_2281	97%	Smlt2233	83%	XCC1854	80%
*rpfB*	AZ78_0629	Long-chain-fatty-acid-CoA ligase	GLE_2284	89%	Smlt2236	79%	XCC1858	74%
*rpfC*	AZ78_3298	Multi-sensor hybrid histidine kinase	GLE_2282	31%	Smlt2234	30%	XCC1856	29%
*rpfF*	AZ78_3297	Enoyl-CoA hydratase	GLE_2283	35%	Smlt2235	34%	XCC1857	35%
*trpC*	AZ78_4108	Indole-3-glycerol phosphate synthase	GLE_4270	87%	Smlt4309	66%	XCC0470	67%
*qseB*	AZ78_2946	Putative two-component response regulator	GLE_5247	93%			XCC3893	59%
*qseC*	AZ78_2945	Two-component system, sensor protein	GLE_5248	82%	Smlt1421	29%	XCC3894	48%
*xanB2*	AZ78_3466	Putative domain	GLE_4979	84%			XCC4014	65%
*lysR*	AZ78_2901	Transcriptional regulator	GLE_5229	90%				
*luxR*	AZ78_4823	Transcriptional activator protein LuxR	GLE_1495	77%	Smlt1839	48%		

*Percent identity at amino acid level between putative cell–cell communication genes of *L. capsici* AZ78 and their orthologues in *L. enzymogenes* C3 (CP013140.1), *S. maltophilia* K279a (AM743169.1), and *X. campestris* pv. *campestris* ATCC 33913^*T*^ (AE008922.1). Corresponding locus tag for each gene and organisms are given.*

### Diffusible Communication Signals Affect *Lysobacter capsici* AZ78 Antimicrobial Activity and Growth

**FIGURE 1:**
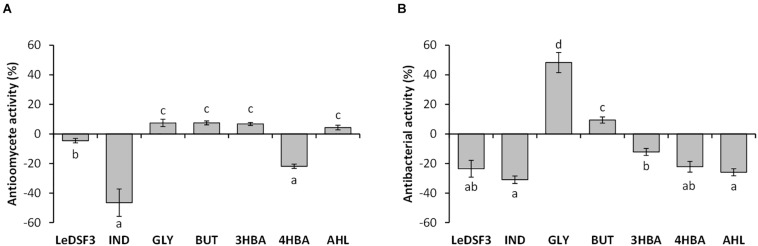
Effect of diffusible communication signals on the inhibitory activity of *Lysobacer capsici* AZ78 against **(A)** *Pythium ultimum* and **(B)** *Rhodococcus fascians*. Antioomycete and antibacterial activities are expressed as the mean value and standard error variation (percentage) of the reduction of the mycelium growth area of *P. ultimum* and *R. fascians* compared with the control (*L. capsici* AZ78 in not supplemented media), respectively. LeDSF3, 13-methyltetradecanoic acid 50 μM; IND, indole 500 μM; GLY, glyoxylic acid 0.01 μM; BUT, 2,3-butanedione 0.01 μM; 3HBA, 3-hydroxybenzoic acid 30 μM; 4HBA, 4-hydroxybenzoic acid 50 μM; AHL, mix of *N*-acyl-homoserine lactones 20 μM. Each treatment included five replicates, and data originating from two independent experiments were pooled. Columns with the same letters are not significantly different according to Tukey’s test (α = 0.05).

Preliminary screening for antimicrobial activity (Supplementary Figure 7) led to the selection of minimum effective concentrations of diffusible communication signals (Supplementary Table 2). Exogenous addition of diffusible communication signals to RMA showed no effect on *P. ultimum* and *R. fascians* LMG 3605 growth, but it modulated AZ78 antimicrobial activity against *P. ultimum* and *R. fascians* LMG 3605 (Figure 1 and Supplementary Figure 7). LeDSF3, 4HBA, and IND decreased AZ78 inhibitory activity against *P. ultimum* by 5, 22, and 47%, respectively. In contrast GLY, BUT, 3HBA, and AHL increased AZ78 inhibitory activity against *P. ultimum* up to 7% (Figure 1). LeDSF3, IND, 3HBA, 4HBA, and AHL decreased AZ78 inhibitory activity against *R. fascians* LMG 3605 up to 31%, while BUT and GLY increased AZ78 antibacterial activity by 9 and 48%, respectively (Figure 1). Changes were particularly relevant for IND and GLY. GLY, BUT, 3HBA, 4HBA, and AHL had no effect on AZ78 growth curves, whereas LeDSF3 and IND slowed down AZ78 growth as compared with the untreated control (Supplementary Figure 8).

### Transcriptional Response of *Lysobacter capsici* AZ78 to Diffusible Communication Signals

The expression of 21% of all AZ78 genes was significantly affected by diffusible communication signals (| log_2_FC| > 1 and *p* < 0.01). The largest number of DEGs, 636 genes (about 11.9% of AZ78 transcriptome), was found upon exposure of AZ78 to LeDSF3 (Table 2). This was followed by IND (603 DEGs, 11.3% of total genes), GLY (292 DEGs, 5.5% of total genes), BUT (237 DEGs, 4.4% of total genes), 3HBA (101 DEGs, 1.9% of total genes), and 4BHA (58 DEGs, 1.1% of total genes). The lowest number of DEGs (24, 0.5% of total genes) occurred among cells treated with AHL (Table 2). RNA-Seq results were validated by the relative expression level of 10 selected genes assessed using qRT-PCR (Supplementary Table 5). A close correlation (Pearson’s *r* = 0.95) was observed between log_2_FC measured with RNA-Seq and qRT-PCR (Supplementary Figure 9).

**TABLE 2:** Differentially expressed genes in *Lysobacter capsici* AZ78 in response to diffusible communication signals after 48-h incubation.

		LeDSF3	IND	GLY	BUT	3HBA	4HBA	AHL
**Upregulated**	*Genes with assigned function*	325	312	142	107	48	34	17
	*Hypothetical proteins*	40	56	42	38	12	14	3
	***Total number of genes***	**365**	**368**	**184**	**145**	**60**	**48**	**20**

**Downregulated**	*Genes with assigned function*	194	169	78	72	33	8	2
	*Hypothetical proteins*	77	66	30	20	8	2	2
	***Total number of genes***	**271**	**235**	**108**	**92**	**41**	**10**	**4**

**Total**	*Genes with assigned function*	519	481	220	179	81	42	19
	*Hypothetical proteins*	117	122	72	58	20	16	5
	***Total number of genes***	**636**	**603**	**292**	**237**	**101**	**58**	**24**

*Each treatment was subjected to pairwise comparison with the untreated control. |Log2-fold change| > 1 and *p*-value < 0.01 were chosen as cut-off values for differential gene expression.*

*LeDSF3, 13-methyltetradecanoic acid 50 μM; IND, indole 500 μM; GLY, glyoxylic acid 0.01 μM; BUT, 2,3-butanedione 0.01 μM; 3HBA, 3-hydroxybenzoic acid 30 μM; 4HBA, 4-hydroxybenzoic acid 50 μM; AHL, mix of *N*-acyl-homoserine lactones 20 μM.*

**FIGURE 2:**
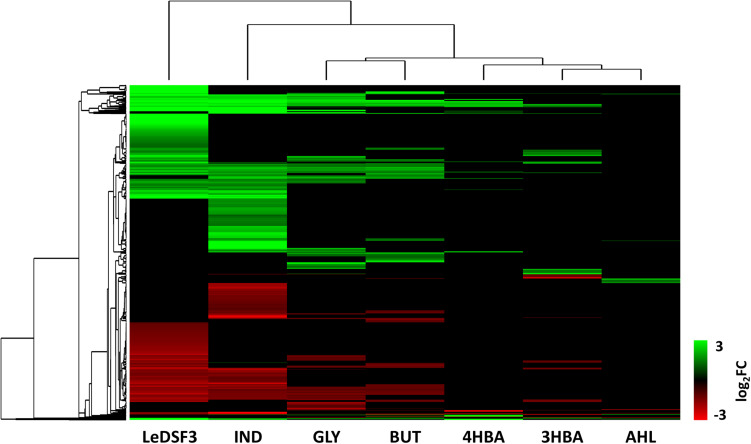
Hierarchical clustering based on log2-fold change (FC) of *Lysobacter capsici* AZ78 genes differentially expressed (1,115 genes, Supplementary Tables 6–12 for gene expression data). LeDSF3, 13-methyltetradecanoic acid 50 μM; IND, indole 500 μM; GLY, glyoxylic acid 0.01 μM; BUT, 2,3-butanedione 0.01 μM; 3HBA, 3-hydroxybenzoic acid 30 μM; 4HBA, 4-hydroxybenzoic acid 50 μM; AHL, mix of *N*-acyl-homoserine lactones 20 μM.

Venn diagrams (Supplementary Figure 10) revealed overlaps among the seven conditions, but they did not identify genes modulated by all seven diffusible communication signals. Moreover, a heatmap (Figure 2) showed that GLY and BUT clustered together, and likewise for 3HBA, 4HBA, and AHL. Instead, LeDSF3 and IND grouped independently.

### The Active Response of *Lysobacter capsici* AZ78 to Diffusible Communication Signals

Functional annotation of AZ78 genes modulated by diffusible communication signals revealed that upregulated DEGs were mainly related to global metabolism; growth; RNA transcription, and degradation; and transport, phosphotransferase systems, and secretion (Table 3 and Supplementary Figure 11). Conversely, downregulated DEGs were mainly related to DNA metabolism.

**TABLE 3:** Number of **(A)** upregulated and **(B)** downregulated differentially expressed genes in *Lysobacter capsici* AZ78 in response to diffusible communication signals.

Class name	IND	LeDSF3	GLY	BUT	3HBA	4HBA	AHL
**(A)**							
Global metabolism	42	34	21	17	2	4	1
Carbohydrate metabolism	17	24	8	4	2	2	0
Energy metabolism	9	17	7	6	6	2	2
Lipid metabolism	5	10	3	2	1	0	0
Nucleotide metabolism	8	11	2	1	1	0	0
Amino acid metabolism	25	29	10	7	5	3	0
Protein metabolism	10	15	10	8	3	1	0
Secondary metabolism	19	8	3	4	2	1	1
DNA metabolism	6	7	3	1	4	0	0
RNA transcription and degradation	37	20	14	14	1	2	4
Translation	14	21	5	2	5	2	0
Growth	28	20	9	10	2	3	1
Oxidative stress	5	3	1	0	1	0	0
Antagonistic activity	9	10	5	3	3	1	1
Defense	13	13	5	3	2	7	7
Transport, phosphotransferase systems, and secretion	35	48	14	11	10	4	7
Signal transduction and receptors	6	7	3	4	1	1	1
Kinase/phosphatase	10	8	5	4	0	0	0
Quorum sensing	1	0	0	0	0	0	1
Motility, chemotaxis, and biofilm	5	11	4	5	1	0	0
Unknown	76	62	54	42	15	17	3
**(B)**							
Global metabolism	12	17	2	7	5	2	0
Carbohydrate metabolism	2	5	3	0	1	0	0
Energy metabolism	12	9	7	7	0	0	0
Lipid metabolism	5	3	1	0	0	0	0
Nucleotide metabolism	1	4	1	0	0	0	0
Amino acid metabolism	3	5	0	2	0	0	0
Protein metabolism	3	5	1	3	2	0	0
Secondary metabolism	1	6	1	0	0	0	0
DNA metabolism	21	23	11	11	1	1	1
RNA transcription and degradation	4	15	3	3	1	1	0
Translation	13	14	18	10	2	0	1
Growth	12	14	6	4	3	0	0
Oxidative stress	1	4	1	0	1	0	0
Antagonistic activity	25	15	5	8	1	0	0
Defense	9	9	5	4	5	0	0
Transport, phosphotransferase systems, and secretion	20	20	5	4	6	3	0
Signal transduction and receptors	3	2	1	2	0	0	0
Kinase/phosphatase	2	3	2	0	0	0	0
Quorum sensing	1	0	0	0	0	0	0
Motility, chemotaxis, and biofilm	7	0	0	0	1	0	0
Unknown	81	100	35	29	13	3	2

*LeDSF3, 13-methyltetradecanoic acid 50 μM; IND, indole 500 μM; GLY, glyoxylic acid 0.01 μM; BUT, 2,3-butanedione 0.01 μM; 3HBA, 3-hydroxybenzoic acid 30 μM; 4HBA, 4-hydroxybenzoic acid 50 μM; AHL, mix of *N*-acyl-homoserine lactones 20 μM.*

LeDSF3 and IND regulated several genes involved in transport, phosphotransferase systems, and secretion; global metabolism; RNA transcription and degradation; and growth (Table 3 and Supplementary Figure 11). In addition, LeDSF3 affected translation and IND antagonism (Table 3 and Supplementary Figure 11). Besides regulating genes related to global metabolism and transport, phosphotransferase systems, and secretion, GLY and BUT modulated a relevant number of genes classified into RNA transcription and degradation and translation, among which tRNA genes were mainly downregulated (Table 3 and Supplementary Figure 11). Genes related to transport, phosphotransferase systems, and secretion and defense were modulated by 4HBA and AHL (Table 3 and Supplementary Figure 11).

**FIGURE 3:**
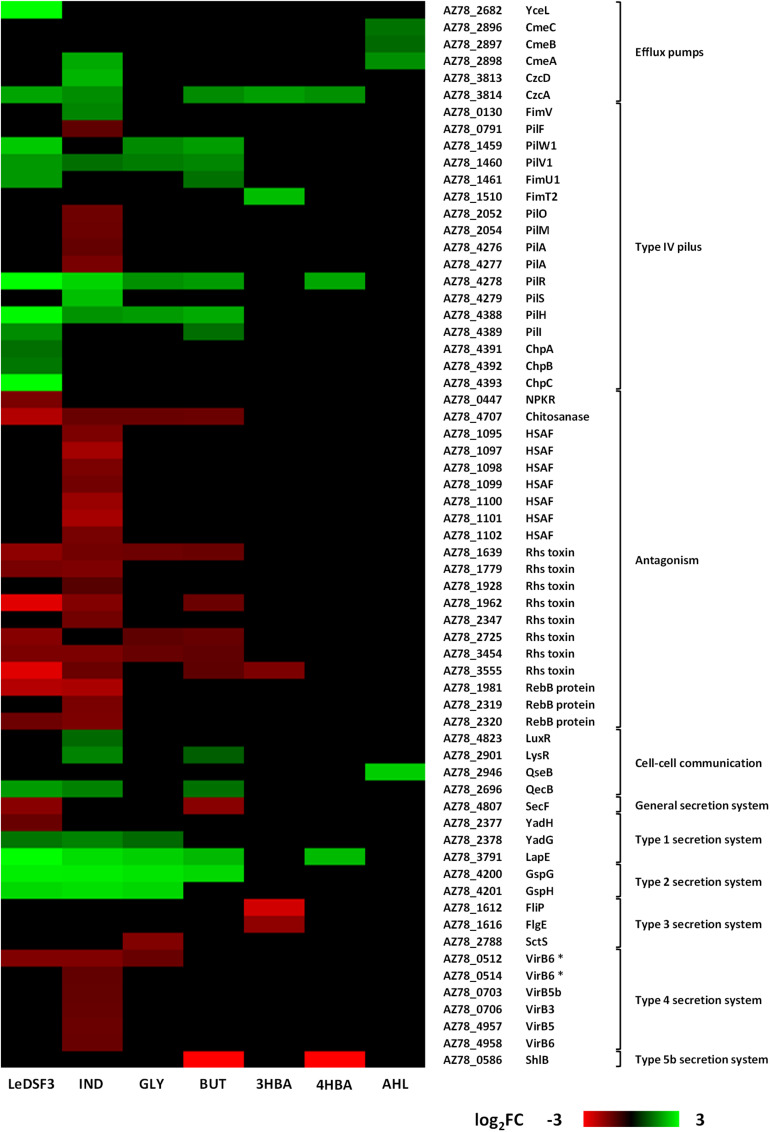
Heatmap based on log2-fold change (FC) of *Lysobacter capsici* AZ78 genes differentially expressed in response to diffusible communication signals and related to efflux pumps, type IV pilus, antagonism, cell–cell communication systems, and secretion systems. HSAF, heat-stable antifungal factor. VirB6 * represents duplicate gene pairs located through AZ78 genome. LeDSF3, 13-methyltetradecanoic acid 50 μM; IND, indole 500 μM; GLY, glyoxylic acid 0.01 μM; BUT, 2,3-butanedione 0.01 μM; 3HBA, 3-hydroxybenzoic acid 30 μM; 4HBA, 4-hydroxybenzoic acid 50 μM; AHL, mix of *N*-acyl-homoserine lactones 20 μM.

Many genes ascribed to transport, phosphotransferase systems, and secretion were involved in drug and metal (particularly iron) transport (Supplementary Tables 6–12). Major facilitator superfamily (MFS) transporters were upregulated by LeDSF3 (Figure 3 and Supplementary Table 6). Resistance–nodulation–division (RND) efflux system genes were upregulated by diffusible communication signals, especially by AHL (Figure 3 and Supplementary Table 12). TonB-dependent receptors involved in the uptake of iron–siderophore complexes or vitamins were upregulated by LeDSF3 and downregulated by IND (Supplementary Tables 6, 7). Additionally, diffusible communication signals upregulated a relevant set of transcription regulators belonging to the AraC, ArsR, TetR, MerR, and MarR families (Supplementary Tables 6–12).

With the only exception of AHL, diffusible communication signals modulated the expression of genes involved in type IV pilus (T4P) biosynthesis. LeDSF3 upregulated the expression of *pilW1-pilV1-fimU1* genes (AZ78_1459–AZ78_1461), related to minor pilins; *pilR* from two-component system *pilR*-*pilS* (AZ78_4278–AZ78_4279); and *pilH* (AZ78_4388), *pilI* (AZ78_4389), *chpA* (AZ78_4391), *chpB* (AZ78_4392), and *chpC* (AZ78_4393) from the pilus-specific chemotaxis system (Pil-Chp) (Figure 3 and Supplementary Table 6). Gene-encoding minor pilins, pilRS, and Pil-Chp were also upregulated in GLY, BUT, and 4HBA (Figure 3 and Supplementary Tables 8, 9, 11). IND upregulated the expression of *fimV* (AZ78_0130), *pilV1*, *pilR*-*pilS*, and *pilH*, while it downregulated *pilF* (AZ78_0791), *pilO* and *pilM* (AZ78_2052 and AZ78_2054), and the major pilin *pilA* (AZ78_4276–AZ78_4277) (Figure 3 and Supplementary Table 7). LeDSF3 and IND downregulated a relevant number of involved in antagonism. IND downregulated the biosynthetic gene cluster AZ78_1095–AZ78_1102, responsible for the production of HSAF (Brescia et al., 2020; Figure 3 and Supplementary Table 7). Moreover, IND upregulated a diguanylate cyclase harboring a GGDEF motif (AZ78_4062) possibly related to cyclic-di-GMP (c-di-GMP) biosynthesis (Supplementary Table 7). Other genes related to antagonism, such as gene-encoding Rhs toxins and RebB proteins, responsible for the expression of killing traits, were downregulated by LeDSF3 and IND (Figure 3 and Supplementary Tables 6, 7).

Diffusible communication signals also caused changes in the expression of genes involved in the reception and regulation of diffusible communication signals in AZ78 (Figure 3). Genes related to Type I secretion system (T1SS) were mostly upregulated by all diffusible communication signals, especially by LeDSF3 and IND (Figure 3 and Supplementary Tables 6, 7). Type II secretion system (T2SS) genes, such as *gspG* (AZ78_4200) and *gspH* (AZ78_4201), were upregulated by LeDSF3, IND, GLY, and BUT (Figure 3 and Supplementary Tables 6–9). On the contrary, the expression of Type III secretion system (T3SS) was downregulated by GLY and 3HBA (Figure 3 and Supplementary Tables 8, 10). Genes associated with type IV secretion system (T4SS) were downregulated by LeDSF3, IND, and GLY (Figure 3 and Supplementary Tables 6–8). ShlB from the two-partner secretion of Type V secretion system (T5bSS) was downregulated by BUT and 4HBA (Figure 3 and Supplementary Tables 9, 11). Finally, LeDSF3, IND, and BUT also regulated general secretory (Sec) pathways (Figure 3 and Supplementary Tables 6, 7, 9).

## Discussion

**FIGURE 4:**
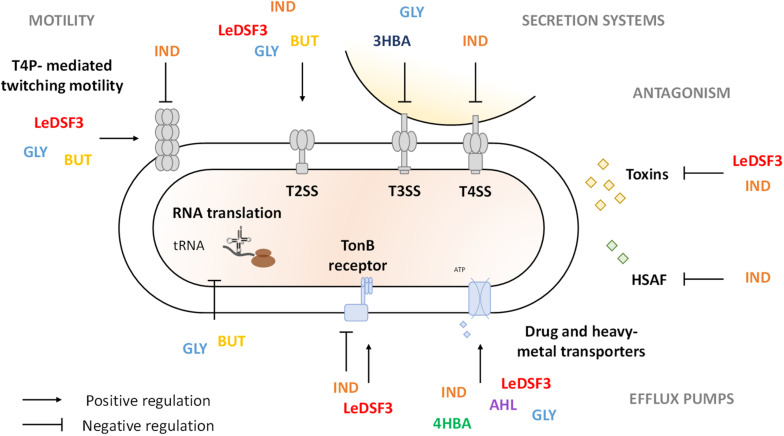
Schematic representation of *Lysobacter capsici* AZ78 response to diffusible communication signals. LeDSF3, 13-methyltetradecanoic acid; IND, indole; GLY, glyoxylic acid; BUT, 2,3-butanedione; 3HBA, 3-hydroxybenzoic acid; 4HBA, 4-hydroxybenzoic acid; AHL, *N*-acyl-homoserine lactones.

The behavior of bacterial species in polymicrobial communities mainly relies on communication systems (Venturi and Keel, 2016); and many secreted metabolites characterizing the cooperation among microorganisms, as well as antibiotics and toxins involved in microbial competition, are controlled by diffusible communication signals (Hibbing et al., 2010; Cornforth and Foster, 2013; Schuster et al., 2013). Diffusible communication signals are involved not only in signaling among self-cells but also in the detection of specific cues produced by other strains or species (Cornforth and Foster, 2013). In fact, many bacterial species have receptors for diffusible communication signals that are not produced by the same species, such as LuxR *solos*, and abundant two-component signaling systems (Cornforth and Foster, 2013). Genome mining results indicate that AZ78 and *Lysobacter* spp. may (at least) produce DSFs, IND, and DFs and perceive DSFs, IND, DFs, and AHLs. As a consequence, diffusible communication signals (mainly IND and GLY) influenced AZ78 antagonistic activity against the phyopathogenic Gram-positive bacteria *R. fascians* and the phyopathogenic oomycete *P. ultimum*. Different intraspecies, interspecies, and interkingdom diffusible communication signals might be used as cues for AZ78 to favor the regulation of molecular pathways related to cell persistence in the rhizosphere or for coercion (Figure 4). Thus, transcriptome profiles showed that diffusible communication signals might contribute to alert AZ78 against toxic compounds produced by other (micro)organisms in the rhizosphere by triggering the expression of gene-encoding efflux pumps that could actively extrude antibiotics, heavy metals, biocides, and solvents (Blanco et al., 2016). In addition, diffusible communication signals might help cells to escape from adverse conditions or to reach nutrients (Chen et al., 2017). For example, LeDSF3, GLY, and BUT upregulated the expression of genes related to the biogenesis of T4P involved in twitching motility. T4P-driven twitching motility is involved in a variety of physiological and social behaviors of a wide range of bacteria (Burrows, 2012; Zhang et al., 2012). For instance, twitching motility is required for colonization and infection of phytopathogenic fungi and oomycetes in *Lysobacter* spp. (Patel et al., 2011; Tomada et al., 2017), and it seems to be a DSF-dependent trait in *L. brunescens* and *L. enzymogenes* (Qian et al., 2013; Feng et al., 2019; Ling et al., 2019b). Interestingly, upregulation of T4P by GLY and BUT came along with increased antimicrobial activity, suggesting that microbial partners producing this kind of diffusible communication signals might encourage AZ78 to form a stable community and stimulate traits responsible for disease suppressiveness in soils. Moreover, GLY and BUT downregulated the transcription of tRNA genes, which might determine a decrease in the tRNA abundance in AZ78 cells. The decrease of tRNA abundance has been already studied in *Escherichia coli* (Potrykus and Cashel, 2008; Zhong et al., 2015). In this bacterial species, the decrease in tRNA abundance was associated with the ability to rapidly adapt to amino acid starvation (Potrykus and Cashel, 2008) and oxidative stress (Zhong et al., 2015). Thus, it is conceivable that the downregulation of tRNA genes in AZ78 cells upon perception of GLY and BUT might contribute to reduce the negative impact of environmental stresses in AZ78 cells. Nevertheless, this hypothesis needs to be proved in future works. In contrast, IND caused a dysregulation of T4P genes in AZ78 with possible losses of T4P functionality. Accordingly, IND decreases motility and biofilm formation in *E. coli* (Domka et al., 2006; Bansal et al., 2007; Lee et al., 2008; Mufti et al., 2015), probably as a manner to save energy and regulate growth dynamics (Nadell et al., 2008). In support of this hypothesis, IND diminished the AZ78 cell growth, although it was not possible to formulate a clear conclusion, as knowledge about IND functions is contrasting (Mueller et al., 2007, 2009; Lee et al., 2009).

In addition, *Lysobacter* cells might contribute to disease suppressiveness of soils by producing extracellular lytic enzymes and antibiotics. Previous findings showed that HSAF biosynthesis is positively regulated by DSF, IND, and 4HBA in *Lysobacter* spp. (Qian et al., 2013; Han et al., 2017; Ling et al., 2019b). Yet in AZ78, IND reduced antioomycete activity and downregulated the expression of the HSAF biosynthetic gene cluster. The downregulation of HSAF related genes by IND might be associated with the simultaneous upregulation of the LuxR *solo* (AZ78_4823), as previously reported in *L. enzymogenes* OH11, where overexpression of *lesR* (LuxR homologue) leads to a decrease in HSAF production (Qian et al., 2014; Xu et al., 2017). Moreover, IND upregulated several transcription regulators—among which various *tetR* repressors (Ramos et al., 2005), like AZ78_0770 and AZ78_3232—that might be involved in HSAF biosynthesis regulation in AZ78, as found for LetR (a TetR family protein) in *L. enzymogenes* OH11 (Wang et al., 2017). The expression of HSAF biosynthetic cluster is also negatively regulated by cyclic-di-GMP (c-di-GMP) in *L. enzymogenes* OH11 (Chen et al., 2017). Interestingly, IND upregulated the expression of a diguanylate cyclase (AZ78_4062) that might be involved in c-di-GMP biosynthesis, implying a regulation role of c-di-GMP in HSAF production in AZ78. Besides producing secondary metabolites with antimicrobial activity, AZ78 might produce diffusible proteinaceous toxins and toxins deployed by contact-dependent systems, such as Rhs toxins, which mediate growth inhibition of neighboring cells in *Dickeya dadantii* (Koskiniemi et al., 2013), or R-bodies, which are responsible for cell membrane disruption and toxins delivery in several bacterial genera (Raymann et al., 2013; Matsuoka et al., 2017). Thus, the downregulation of several *rhs* and *reb* genes required for Rhs toxins and R-bodies synthesis by LeDSF3 and IND might have contributed to lower AZ78 the antioomycete and antibacterial activities. Moreover, AZ78 downregulated signal transduction pathways in the presence of IND, such as TonB-dependent receptors, which play a key role in microbial competition with the uptake of iron–siderophore complex or vitamins (Braun, 1995).

Bacteria often use secretion systems to manipulate and kill rival bacterial and eukaryotic cells (Tseng et al., 2009; Green and Mecsas, 2016). Of those, T3SS, T4SS, and T6SS are related to the establishment of pathogenic interactions with microbial hosts in *Lysobacter* spp. (de Bruijn et al., 2015; Yang et al., 2020; Shen et al., 2021). Thus, modulation of genes related to secretion systems might result in gain/loss of ability to compete with other (micro)organisms (Ling et al., 2019a). In agreement with this statement, IND downregulated T4SS and decreased antimicrobial activity in AZ78. Downregulation of T4SS by IND might be related to the overexpression of diguanylate cyclases (e.g., AZ78_4062), responsible for c-di-GMP increase and T4SS inactivation in *A. tumefaciens* (McCarthy et al., 2019). However, T3SS was downregulated by GLY and 3HBA with no decrease in AZ78 toxic activity, suggesting that it was probably repressed to save energy under conditions where it does not provide an advantage, as found in *Pseudomonas aeruginosa* (Bleves et al., 2005), *Vibrio harveyi* (Ruwandeepika et al., 2015), and *Yersinia pseudotuberculosis* (Atkinson et al., 2011).

## Conclusion

Overall, functional and transcriptome analysis of AZ78 shed light on the key role of signaling communication systems on the recruiting and shaping of AZ78 in the rhizosphere. Our results show that GLY and BUT might facilitate AZ78 rhizosphere competence and soil suppressiveness to plant pathogens. On the other hand, IND might prevent AZ78 from growing at high cell densities and decrease motility. Moreover, IND and LeDSF3 might decrease AZ78 ability to control phytopathogenic microorganisms. Manipulating diffusible communication signals levels in the rhizosphere could therefore provide efficient means to favor persistence and functioning of specific groups of beneficial bacteria, such as *Lysobacter* strains, at the root–soil interface.

## Data Availability Statement

The datasets presented in this study can be found in online repositories. The names of the repository/repositories and accession number(s) can be found below: https://www.ncbi.nlm.nih.gov/, PRJNA714393.

## Author Contributions

AB and GP conceived the study, performed the experiments, analyzed the data, and conceptualized and drafted the manuscript. MP helped in the experimental setup, provided input, and proofread the manuscript. IP provided input and proofread the manuscript. All authors contributed to the article and approved the submitted version.

## Conflict of Interest

The authors declare that the research was conducted in the absence of any commercial or financial relationships that could be construed as a potential conflict of interest.

## Publisher’s Note

All claims expressed in this article are solely those of the authors and do not necessarily represent those of their affiliated organizations, or those of the publisher, the editors and the reviewers. Any product that may be evaluated in this article, or claim that may be made by its manufacturer, is not guaranteed or endorsed by the publisher.

## Footnotes

1
https://www.ncbi.nlm.nih.gov/

2
www.biobam.com/omicsbox

3
http://www.blast2go.org

## Supplementary Material

Supplementary Figure 1 Neighbor-joining trees illustrating the relationships across *Lysobacter* members based on nucleotide sequences of the *rpfG* and *rpfB* genes. *Pseudomonas aeruginosa* PAO1 was used as outgroup sequence. Locus tag numbers are given in brackets, GenBank accession numbers for the whole genome sequences are given in Supplementary Table 1.

Supplementary Figure 2 Neighbor-joining trees illustrating the relationships across *Lysobacter* members based on nucleotide sequences of the *rpfC* and *rpfF* genes. *Pseudomonas aeruginosa* PAO1 was used as outgroup sequence. Locus tag numbers are given in brackets, GenBank accession numbers for the whole genome sequences are given in Supplementary Table 1.

Supplementary Figure 3 Location of *rpf* genes in *Lysobacter capsici* AZ78. **(a)** Comparison of the clusters of genes involved in Diffusible Soluble Factors synthesis and perception in *L. enzymogenes* C3 and *L. capsici* AZ78. Gene names are given within the arrows. The corresponding accession number is given under each gene. **(b)** Bioassay showing DSF production ability (blue halo) of *L. capsici* AZ78 in comparison to *Xanthomonas campestris* pv. *campestris* 8004.

Supplementary Figure 4 Neighbor-joining trees illustrating the relationships across *Lysobacter* members based on nucleotide sequences of the *tprC, qseB*, and *qseC* genes. *Burkholderia cenocepacia* KC-01 or *Pseudomonas aeruginosa* PAO1 were used as outgroup sequence. Locus tag numbers are given in brackets, GenBank accession numbers for the whole genome sequences are given in Supplementary Table 1.

Supplementary Figure 5 Neighbor-joining trees illustrating the relationships across *Lysobacter* members based on nucleotide sequences of the *xanB2, lysR*, and *luxR* genes. *Pseudomonas aeruginosa* PAO1 was used as outgroup sequence. Locus tag numbers are given in brackets, GenBank accession numbers for the whole genome sequences are given in Supplementary Table 1.

Supplementary Figure 6 Bioassay of *N*-acyl-homoserine lactones. *N*-acyl-homoserine lactones are produced by *Lysobacter daejeonensis* GH1-9^*T*^ (purplish color in the reporter strain *Chromobacterium violaceum* CV026), but not by *L. capsici* AZ78.

Supplementary Figure 7 Effect of diffusible communication signals on the inhibitory activity of *Lysobacter capsici* AZ78 against **(a–f)** *Pythium ultimum* and **(g–l)** *Rhodococcus fascians*. Antioomycete and antibacterial activity is expressed as the mean value and standard error variation (percentage) of the reduction of the mycelium growth area of *P. ultimum* and *R. fascians* compared to the control (*L. capsici* AZ78 in not supplemented media), respectively. **(a,g)** 13-methyltetradecanoic acid, **(b,h)** glyoxylic acid, **(c,i)** 2,3-butanedione, **(d,j)** 3-hydroxybenzoic acid, **(e,k)** 4-hydroxybenzoic acid, **(f,l)** mix of *N*-acyl-homoserine lactones. Each treatment included five replicates and data originating from two independent experiments were pooled. Different letters indicate significant differences according to Tukey’s test (α = 0.05). Eventual minimum effective concentrations are given in Supplementary Table 2.

Supplementary Figure 8 Growth curves of *Lysobacter capsici* AZ78 exposed to diffusible communication signals. LeDSF3, 13-methyltetradecanoic acid 50 μM; IND, indole 500 μM; GLY, glyoxylic acid 0.01 μM; BUT, 2,3-butanedione 0.01 μM; 4-HBA, 4-hydroxybenzoic acid 50 μM; 3-HBA, 3-hydroxybenzoic acid 30 μM; AHL, mix of *N*-acyl-homoserine lactones 20 μM; TSB, 1/10 Tryptic Soy Broth; MeOH, 1% v/v methanol.

Supplementary Figure 9 Scatter plot of RNA-Seq and qRT-PCR relative expression levels. Pearson correlation test (*r* = 0.95) was applied to log2 fold change (FC) values of selected genes (Supplementary Table 4).

Supplementary Figure 10 Venn diagram of up-regulated **(a)** and down-regulated **(b)** genes indicating the overlap in the number of differentially expressed genes (DEGs) in *Lysobacter capsici* AZ78 as response to diffusible communication signals. Only genes with |log2-fold change| > 1 and *p*-value < 0.01 were included. LeDSF3, 13-methyltetradecanoic acid 50 μM; IND, indole 500 μM; GLY, glyoxylic acid 0.01 μM; BUT, 2,3-butanedione 0.01 μM; 4-HBA, 4-hydroxybenzoic acid 50 μM; 3-HBA, 3-hydroxybenzoic acid 30 μM; AHL, mix of *N*-acyl-homoserine lactones 20 μM.

Supplementary Figure 11 Radar plots of *Lysobacter capsici* AZ78 genes differentially expressed in response to diffusible communication signals. Annotated genes were classified in 20 functional categories: global metabolism (MET); carbohydrate metabolism (CM); energy metabolism (E); lipid metabolism (LM); nucleotide metabolism (NM); amino acid metabolism (AM); protein metabolism (PM); secondary metabolism (SM); DNA metabolism (DNA); RNA transcription and degradation (RNA/TR); translation (T); growth (G); oxidative stress (OX); antagonism (AG); defence (D); transport, phosphotransferase systems, and secretion (T); signal transduction and receptors (S); kinase/phosphatase (K); quorum sensing (QS); motility, chemotaxis, and biofilm (M). Only genes with |log2-fold change| > 1 and *p*-value < 0.01 were included. LeDSF3, 13-methyltetradecanoic acid 50 μM; IND, indole 500 μM; GLY, glyoxylic acid 0.01 μM; BUT, 2,3-butanedione 0.01 μM; 3HBA, 3-hydroxybenzoic acid 30 μM; 4HBA, 4-hydroxybenzoic acid 50 μM; AHL, mix of *N*-acyl-homoserine lactones 20 μM.

Supplementary Table 1 Bacterial strains used for phylogenetic analysis.

Supplementary Table 2 Culture conditions of *L. capsici* AZ78.

Supplementary Table 3 RNA integrity and concentration of *Lysobacter capsici* AZ78 samples.

Supplementary Table 4 Number of reads obtained from Illumina HiSeq sequencing of RNA extracted from different *Lysobacter capsici* AZ78.

Supplementary Table 5 Primers used in qRT-PCR.

Supplementary Table 6 List of differentially expressed genes of *Lysobacter capsici* AZ78 after 48 h incubation with 13-methyltetradecanoic acid 50 μM.

Supplementary Table 7 List of differentially expressed genes of *Lysobacter capsici* AZ78 after 48 h incubation with indole 500 μM.

Supplementary Table 8 List of differentially expressed genes of *Lysobacter capsici* AZ78 after 48 h incubation with glyoxylic acid 0.01 μM.

Supplementary Table 9 List of differentially expressed genes of *Lysobacter capsici* AZ78 after 48 h incubation with 2,3-butanedione 0.01 μM.

Supplementary Table 10 List of differentially expressed genes of *Lysobacter capsici* AZ78 after 48 h incubation with 3-hydroxybenzoic acid 30 μM.

Supplementary Table 11 List of differentially expressed genes of *Lysobacter capsici* AZ78 after 48 h incubation with 4-hydroxybenzoic acid 50 μM.

Supplementary Table 12 List of differentially expressed genes of *Lysobacter capsici* AZ78 after 48 h incubation with mix of *N*-acyl-homoserine lactones 20 μM.
