# Supplementary material for: The Perception of Rhizosphere Bacterial Communication Signals Leads to Transcriptome Reprogramming in Lysobacter capsici AZ78, a Plant Beneficial Bacterium
Source: Front Microbiol. 2021 Aug 18;12:725403. doi: 10.3389/fmicb.2021.725403 (PMC8416617; doi:10.3389/fmicb.2021.725403)
Supplement: Supplementary Figure 1 — Neighbor-joining trees illustrating the relationships across Lysobacter members based on nucleotide sequences of the rpfG and rpfB genes. Pseudomonas aeruginosa PAO1 was used as outgroup sequence. Locus tag numbers are given in brackets, GenBank accession numbers for the whole genome sequences are given in Supplementary Table 1. [file Data_Sheet_1.docx]

**Supplementary material**

**Supplementary figures**

Supplementary Figure 1. Neighbor-joining trees illustrating the relationships across *Lysobacter* members based on nucleotide sequences of the *rpfG* and *rpfB* genes. *Pseudomonas aeruginosa* PAO1 was used as outgroup sequence. Locus tag numbers are given in brackets, GenBank accession numbers for the whole genome sequences are given in Supplementary Table 1.

Supplementary Figure 2. Neighbor-joining trees illustrating the relationships across *Lysobacter* members based on nucleotide sequences of the *rpfC* and *rpfF* genes. *Pseudomonas aeruginosa* PAO1 was used as outgroup sequence. Locus tag numbers are given in brackets, GenBank accession numbers for the whole genome sequences are given in Supplementary Table 1.

Supplementary Figure 3. Location of *rpf* genes in *Lysobacter capsici* AZ78. (a) Comparison of the clusters of genes involved in Diffusible Soluble Factors synthesis and perception in *L. enzymogenes* C3 and *L. capsici* AZ78. Gene names are given within the arrows. The corresponding accession number is given under each gene. (b) Bioassay showing DSF production ability (blue halo) of *L. capsici* AZ78 in comparison to *Xanthomonas campestris* pv. *campestris* 8004.

Supplementary Figure 4. Neighbor-joining trees illustrating the relationships across *Lysobacter* members based on nucleotide sequences of the *tprC, qseB,* and *qseC* genes. *Burkholderia cenocepacia* KC-01 or *Pseudomonas aeruginosa* PAO1 were used as outgroup sequence. Locus tag numbers are given in brackets, GenBank accession numbers for the whole genome sequences are given in Supplementary Table 1.

Supplementary Figure 5. Neighbor-joining trees illustrating the relationships across *Lysobacter* members based on nucleotide sequences of the *xanB2, lysR,* and *luxR* genes. *Pseudomonas aeruginosa* PAO1 was used as outgroup sequence. Locus tag numbers are given in brackets, GenBank accession numbers for the whole genome sequences are given in Supplementary Table 1.

Supplementary Figure 6. Bioassay of N-acyl-homoserine lactones. N-acyl-homoserine lactones are produced by *Lysobacter daejeonensis* GH1-9^T^ (purplish colourin the reporter strain*Chromobacterium violaceum* CV026), but not by *L. capsici*AZ78.

Supplementary Figure 7. Effect of diffusible communication signals on the inhibitory activity of *Lysobacter capsici* AZ78 against (a-f) *Pythium ultimum* and (g-l) *Rhodococcus fascians*. Antioomycete and antibacterial activity is expressed as the mean value and standard error variation (percentage) of the reduction of the mycelium growth area of *P. ultimum* and *R. fascians* compared to the control (*L. capsici* AZ78 in not supplemented media), respectively. (a, g) 13-methyltetradecanoic acid, (b, h) glyoxylic acid, (c, i) 2,3-butanedione, (d, j) 3-hydroxybenzoic acid, (e, k) 4-hydroxybenzoic acid, (f, l) mix of N-acyl homoserine lactones. Each treatment included five replicates and data originating from two independent experiments were pooled. Different letters indicate significant differences according to Tukey’s test (α = 0.05). Eventual minimum effective concentrations are given in Supplementary Table 2.

Supplementary Figure 8. Growth curves of *Lysobacter capsici* AZ78 exposed to diffusible communication signals. LeDSF3: 13-methyltetradecanoic acid 50 µM, IND: indole 500 µM, GLY: glyoxylic acid 0.01 µM, BUT: 2,3-butanedione 0.01 µM, 4-HBA: 4-hydroxybenzoic acid 50 µM, 3-HBA: 3-hydroxybenzoic acid 30 µM, AHL: mix of N-acyl homoserine lactones 20 µM, TSB: 1/10 Tryptic Soy Broth, MeOH: 1% v/v methanol.

Supplementary Figure 9. Scatter plot of RNA-Seq and qRT-PCR relative expression levels. Pearson correlation test (*r* = 0.95) was applied to log2 fold change (FC) values of selected genes (Supplementary Table 4).

Supplementary Figure 10. Venn diagram of up-regulated (a) and down-regulated (b) genes indicating the overlap in the number of differentially expressed genes (DEGs) in *Lysobacter capsici* AZ78 as response to diffusible communication signals. Only genes with |log2-fold change| > 1 and p-value < 0.01 were included. LeDSF3: 13-methyltetradecanoic acid 50 µM, IND: indole 500 µM, GLY: glyoxylic acid 0.01 µM, BUT: 2,3-butanedione 0.01 µM, 4-HBA: 4-hydroxybenzoic acid 50 µM, 3-HBA: 3-hydroxybenzoic acid 30 µM, AHL: mix of N-acyl homoserine lactones 20 µM.

Supplementary Figure 11. Radar plots of *Lysobacter capsici* AZ78 genes differentially expressed in response to diffusible communication signals. Annotated genes were classified in 20 functional categories: global metabolism (MET); carbohydrate metabolism (CM); energy metabolism (E); lipid metabolism (LM); nucleotide metabolism (NM); amino acid metabolism (AM); protein metabolism (PM); secondary metabolism (SM); DNA metabolism (DNA); RNA transcription and degradation (RNA/TR); translation (T); growth (G); oxidative stress (OX); antagonism (AG); defence (D); transport, phosphotransferase systems and secretion (T); signal transduction and receptors (S); kinase/phosphatase (K); quorum sensing (QS); motility, chemotaxis, and biofilm (M). Only genes with |log2-fold change| > 1 and p-value < 0.01 were included. LeDSF3: 13-methyltetradecanoic acid 50 µM, IND: indole 500 µM, GLY: glyoxylic acid 0.01 µM, BUT: 2,3-butanedione 0.01 µM, 3HBA: 3-hydroxybenzoic acid 30 µM, 4HBA: 4-hydroxybenzoic acid 50 µM, AHL: mix of N-acyl homoserine lactones 20 µM.

**Supplementary tables in the Excel file**

Supplementary Table 1. Bacterial strains used for phylogenetic analysis.

Supplementary Table 2. Culture conditions of *L. capsici* AZ78.

Supplementary Table 3.RNA integrity and concentration of *Lysobacter capsici* AZ78 samples.

Supplementary Table 4. Number of reads obtained from Illumina HiSeq sequencing of RNA extracted from different *Lysobacter capsici* AZ78.

Supplementary Table 5. Primers used in qRT-PCR.

Supplementary Table 6. List of differentially expressed genes of *Lysobacter capsici* AZ78 after 48 h incubation with 13-methyltetradecanoic acid 50 µM.

Supplementary Table 7. List of differentially expressed genes of *Lysobacter capsici* AZ78 after 48 h incubation with indole 500 µM.

Supplementary Table 8. List of differentially expressed genes of *Lysobacter capsici* AZ78 after 48 h incubation with glyoxylic acid 0.01 µM.

Supplementary Table 9. List of differentially expressed genes of *Lysobacter capsici* AZ78 after 48 h incubation with 2,3-butanedione 0.01 µM.

Supplementary Table 10. List of differentially expressed genes of *Lysobacter capsici* AZ78 after 48 h incubation with 3-hydroxybenzoic acid 30 µM.

Supplementary Table 11. List of differentially expressed genes of *Lysobacter capsici* AZ78 after 48 h incubation with 4-hydroxybenzoic acid 50 µM.

Supplementary Table 12. List of differentially expressed genes of *Lysobacter capsici* AZ78 after 48 h incubation with mix of N-acyl homoserine lactones 20 µM.
